# Women’s vulnerability within the childbearing continuum: A scoping review

**DOI:** 10.18332/ejm/120003

**Published:** 2020-05-12

**Authors:** Elisabetta Colciago, Beatrice Merazzi, Maria Panzeri, Simona Fumagalli, Antonella Nespoli

**Affiliations:** School of Medicine and Surgery, University of Milano-Bicocca, Monza, Italy; Foundation MBBM, Monza, Italy

**Keywords:** pregnant women, vulnerability, midwifery care

## Abstract

**INTRODUCTION:**

The aim of this scoping review is to explore the concept of ‘vulnerability’ affecting pregnant women and to identify an appropriate definition of this term.

**METHODS:**

Five stages were adopted for conducting the scoping review. A literature search was undertaken between 1 October 2017 and 5 January 2018, using three databases. Relevant publications were appraised, and semantic content analysis was performed to identify emergent themes and four determinants of the vulnerability concept. This involved combining items that seem to address the same issue.

**RESULTS:**

Eleven publications were considered, and eight definitions of vulnerability were identified, and from these four themes emerged: poor health outcome or status; exposure to risk; complex social needs; and lack of resources. Further analysis of evidence found examples of groups of people considered to be vulnerable; they were reported into six matrices, mainly with social and psychological difficulties. From these, eleven themes arose. Following a semantic and content analysis of all themes, thirteen final themes were identified. They represent the characteristics associated with women considered to be vulnerable and are called indices of vulnerability. Semantic and content analysis allowed addressing the thirteen indices of vulnerability into four categories called determinants of the vulnerability concept: deficiency, need, risk exposure, and barriers.

**CONCLUSIONS:**

The vulnerability could be defined as a lack of health, related to the presence of at least one of the four determinants. Midwives are the key to identify vulnerable women, offering appropriate care.

## INTRODUCTION

Different factors, called Determinants of Health^1^, affect the health of individuals and communities and create different living conditions that impact on health. They include biological aspects, combined with income and social status, education, employment and working conditions, access to appropriate health services, and the physical environments. Difficulties experienced in one of the areas represented by the Determinants of Health could pose a potential ‘risk’ affecting maternal and neonatal outcomes^2-4^. Almost everywhere in the world, girls and women living in wealthier households have higher use of healthcare services than those living in the poorest families, leading to lower levels of mortality^5^. Such differences are not confined to developing countries but are also found in the developed world^5^, where many marginalised subpopulations continue to experience a range of inequities in maternal health^6^. Great diversity in cultural plus social, financial and linguistic factors lead to potentially worse perinatal outcomes^7^; thus social aspects are major determinants of perinatal health^7^.

The context in which a woman lives is a relevant factor and should be considered while delivering midwifery care. A scoping review by Khanlou et al.^8^, reported that countries of the European Union present several factors such as financial and linguistic barriers, lack of antenatal care and immigration, which lead to pregnancy-related problems, higher rates of stillbirths and infant mortality. Women with these social issues are more likely to be considered vulnerable and would need midwifery care accordingly^9^. Some other risk factors associated with perinatal health, such as older age at childbirth or maternal obesity, are increasing in all countries^7^. Furthermore, survey data show that women in all countries are subject to violence. One in five women has been a victim of domestic violence^10,11^, which is associated with significantly increased risk of low birthweight and preterm birth^7^. The results of a scoping review by Grabovschi et al.^12^ suggest that high levels of vulnerability (defined as multiple vulnerability aspects) would increase healthcare needs and would be associated with lower healthcare accessibility and quality. Midwives have a special role to play in the care of vulnerable women and prevention or mitigation of poor outcomes related to their vulnerability. Evidence^13^ suggests that midwife-led model in women of low socioeconomic position is associated with lower odds of small for gestational age and preterm births and low birthweight babies, compared to physician or obstetrician models of care. Therefore, there needs to be a clear definition of what makes women vulnerable in the context of pregnancy and maternal/newborn outcomes. This work seeks to review the literature on vulnerability and maternal/newborn outcomes to develop a clear definition of vulnerability in pregnancy that includes the leading socioeconomic risk factors correlated to poor outcomes.

## METHODS

The aim of a scoping review is to address an exploratory research question aimed at mapping key concepts, types of evidence, and gaps in research related to a defined area or field by systematically searching, selecting, and synthesising existing knowledge^14^. The overall aim of this work is to provide an overview of a broad topic, which is the concept of vulnerability affecting pregnant women, to capture a wide range of study designs and to identify areas that need further research. A greater understanding of existing definitions of vulnerability and factors that could lead to a status of vulnerability will be presented in order to improve the identification of a vulnerable population. The literature review examining the concept of vulnerability indicated there were limited research and limited primary studies on this topic; therefore the scoping review was considered an appropriate methodology to explore the current knowledge of the topic.

This study adopted the methodology for a scoping review suggested by Arksey and O’Malley^15^ and the advanced methodology developed by Levac et al.^16^. Arksey and O’Malley^15^ indicated four reasons for undertaking a scoping review: 1) to examine the extent, range and nature of research activity, 2) to determine the value of undertaking a full systematic review, 3) to summarise and disseminate research findings, and 4) to identify research gaps in the existing literature. The six stages of the methodological framework adopted to conduct this scoping, as suggested by Arksey and O’Malley^15^, were: 1) identifying the research question; 2) identifying relevant studies; 3) study selection; 4) charting the data; 5) collating, summarising and reporting the results; and 6) consultation exercise (optional), which was not undertaken as there is a lack of clarity about timing and on how to integrate the information with study findings^16^. For the fifth stage the methodology developed by Levac et al.^16^ was adopted. The scoping review’s stages are presented below.

### Stage one

The research question of this study was: ‘What is the meaning of the term vulnerability when it is associated with women during pregnancy, birth and the postnatal period and what is the appropriate definition of it?’.

The purpose of this scoping review was to explore the concept of vulnerability and to identify a clear definition of vulnerability into the midwifery area.

### Stage two

A literature search was undertaken using PubMed, Cinahl and Scopus, between 1 October 2017 and 5 January 2018. The following search terms were used, using Boolean operators and according to the index system of each database: [Vulnerability] AND [Midwifery OR Midwife]; [Vulnerability] AND [Pregnancy OR Gestation OR Gravidity]; [Vulnerable pregnancy]; [Vulnerability] AND [Index OR Factor OR Indices] AND [Pregnancy].

The review considered English language studies from 1 January 1990 and onwards. No limits were imposed on the study’s design or regarding primary or secondary research articles.

### Stage three

The search generated 6829 articles. Duplicates were eliminated, articles were evaluated by reading the title and abstract by two reviewers. Eighty-seven studies were relevant to the search question. Articles were then evaluated by reading the full text, and nine articles were included in the scoping review. Discussion among the midwifery research team led to a consensus about articles considered.

A manual search was also performed and the following publications were included: one National Institute for Health and Clinical Excellence (NICE) Guideline and one Guidance of the Glasgow City Council. Eleven articles were included in the final review.

### Stage four

The data charted are shown in Table 1, and general information about the articles found are reported including: authors, year of publication, methodology of the study, aim of the study, and important findings. Discussion among researchers allowed establishing the themes that emerged from the content of articles.

**Table 1:** Literature included in the scoping review

*Article and Year*	*Methodology*	*Aim*	*Findings*
National Institute for Health and Clinical Excellence (NICE)^9^ 2010	Clinical guideline	This guideline sets out recommendations for healthcare professionals to address vulnerable women’s needs and to improve pregnancy outcomes in this population.	The NICE guideline identified four groups of vulnerable women: pregnant women who misuse alcohol or drugs, pregnant women who are recent migrants, asylum seekers or who have difficulty reading or speaking English, young pregnant women aged <20 years and pregnant women who experienced domestic abuse. This guideline gives recommendations in order to improve access to care and to offer proper additional care to pregnant women with complex social factors.
Scupholme et al.^18^ 1992	Quantitative survey	To describe the extent to which certified nursemidwives (CNMs) provide care to vulnerable populations in the United States and the source of reimbursement for this care.	Ninety-nine per cent of CNMs in all types of practices report providing care at least to one group of vulnerable women, and CNMs in the inner city and rural practices serve several groups. The vast majority of CNMs are salaried; 11% receive their primary income from fee-for-service, 50% from Medicaid and government-subsidised sources, less than 20% from private insurance. CNMs make a major contribution to the care of vulnerable populations.
Menke et al.^19^ 2014	Qualitative, descriptive phenomenology	To examine midwives’ perceptions of organisational structures and processes of care when working in a caseload model for socially disadvantaged and vulnerable childbearing women.	The study demonstrated that midwives were adept at responding to the diverse needs of women with a wide range of risk profiles. The research found that midwives perceived they could make a difference in women’s lives after birth. Receiving caseload care was viewed as a potentially transformative journey for many women and impacted on the women’s lives in positive ways. Midwives felt that relationships with other members of the healthcare team were typified by lack of respect, minimal collaboration, and the imposition of clinical practices that were perceived by participating midwives to be ‘outdated’ rather than based on best available evidence.
Glasgow Child Protection Committee^20^ 2008	Procedural guidance	To assist vulnerable parents to acquire the necessary parenting skills.	This guidance identified categories of women who require interagency support. The guidance recommends the identification of vulnerable mothers, to assess their needs and the potential risks of the unborn child, in order to put in place appropriate services.
de Groot et al.^21^ 2016	Quantitative and qualitative, mixed method design	To investigate whether the subjective caregiver’s perception of workload and the objective registry-based caseload of vulnerable clients are in agreement, and whether a structure organisation of antenatal risk management reduces the burden associated with perceived workload, in particular if the objective caseload is high.	This study addressed the effect of a specific antenatal practice setting on the subjective workload and associated burden of vulnerable clients, in a region with multiple deprivation areas. Subjective workload and objective caseload were only weakly related, the relation being modified by the organisation of antenatal risk management. If the organisational structure of antenatal risk management was low, the experienced burden was high, even if the objective caseload was low. Highly structured antenatal risk management was associated with medium to low burden. Study suggests that changing antenatal risk management practice policies towards more structured care provision not only may benefit vulnerable clients and their offspring, but also may benefit the healthcare providers in work satisfaction. Increased prevalence of vulnerable clients induces an increased strain on midwives, obstetricians and other healthcare professionals involved in antenatal care.
Briscoe et al.^22^ 2016	Concept analysis	To develop a concept analysis to identify how the term vulnerability is currently understood and used in relation to pregnancy, birth and the postnatal period.	Vulnerability should be viewed as a complex phenomenon. It can be defined by three main attributes, which are: Threat, Barrier and Repair. These attributes could have an impact on maternal outcomes. Subattributes as attachment between mother and baby, woman’s free will and choice added complexity to the concept.
MacMullen et al.^23^ 1992	Literature review	To describes stress factors related to vulnerability in pregnancy and the implementation of a support group as one intervention able to reduce the vulnerability in a group of women during the antenatal period	Women with high risk pregnancies could be hospitalised during the antenatal period. Women could be exposed to vulnerability due to the psychological and physiological disruptions that accompany hospitalisation, leading to increase anxiety and stress. Authors identified twelve stressor themes that contribute to antenatal vulnerability. They described the implementation of a support group, that lasted for over a year, with the aim to reduce the vulnerability in a group of pregnant women.
Tezcan et al.^24^ 2011	Quantitative survey	To assess the feasibility of using a mobile text to reach vulnerable pregnant or postnatal women.	The mobile technology is readily available for 97% of the population considered. Of 94 women who responded, 28% (n=26) admitted to having forgotten at least one antenatal or scan appointment, while 21% (n=20) missed an appointment because they had not received a letter. The majority (61%) of women who were from vulnerable groups or from deprived areas, possessed third generation mobile technology. The survey showed that a significant majority of women would like to have reminders via mobile about appointments and medication taking. These requests for mobile information are even higher in the more vulnerable proportion of the study population.
White et al.^25^ 2015	Quantitative non-randomized controlled study	To assess the efficacy of a primarily antenatal intervention with a traditional hard-to-reach population.	Intervening in the antenatal period may improve outcomes for pregnant women with additional health and social-care needs and their infants, and be more cost-effective than intervening later. Results suggested that psycho-educational antenatal interventions may benefit pregnant women with significant psychosocial needs. Further research with a larger sample size is required.
Malebranche et al.^26^ 2017	Discussion paper, editorial	To discuss the importance of addressing specific mental, physical and reproductive health needs of refugee women, especially during pregnancy	Studies that have explored health outcomes among resettled refugee women demonstrated significant disparities in maternal and perinatal outcomes. Adverse outcomes included higher rates of preterm birth, low birthweight infants, stillbirths and maternal mortality. These could have long-lasting impact on the health and development of the newborn, well into adulthood. High income countries should take action, offering equal opportunity and interventions especially during pregnancy, that minimise the difference between the local population and the refugee one.
Birtwell et al.^28^ 2015	Qualitative, interpretive phenomenology	To understand the experiences of pregnancy for a group of vulnerable women and to understand their experiences following an intervention (called Mellow Bumps) designed to address some of their vulnerabilities.	Authors identified 5 superordinate and 14 master themes; each master theme was divided into multiple subthemes. The study demonstrated a significant overlap between vulnerable women and ‘ordinary’ women’s experiences of pregnancy. Furthermore, authors endorsed the notion that the period of pregnancy may provide a unique and optimal opportunity to intervene to effect change at the level of prenatal attachment, with possible subsequent benefits for longer term postnatal attachment.

### Stage five

As mentioned above, the methodology by Levac et al.^16^ was adopted to collate, summarise and report findings. This methodology comprises three distinct steps: 1) Conduct a descriptive numerical summary analysis and qualitative thematic analysis; 2) Report the results and produce the outcome that refers to the overall purpose or research question; and 3) Consider the meaning of the findings as they relate to the overall study purpose and discuss implications for future research, practice and policy.

Content and semantic analysis was adopted to identify emergent themes from the evidence considered into this scoping review. This is a process to accurately reflect the meaning of concepts and organising information that seems to address the same issue^17^. The first step of the analysis allowed collecting the definitions of vulnerability and the second step involved combining examples of vulnerable people. During the analysis of both steps, the researchers looked for commonalities, differences and associations, and identifying potential emergent themes. Lastly, semantic and content analysis focused in organising a group of repeating ideas to find the determinants of the vulnerability concept; these were adjusted until agreement on final findings was achieved by all members of the team.

**Figure 1:**
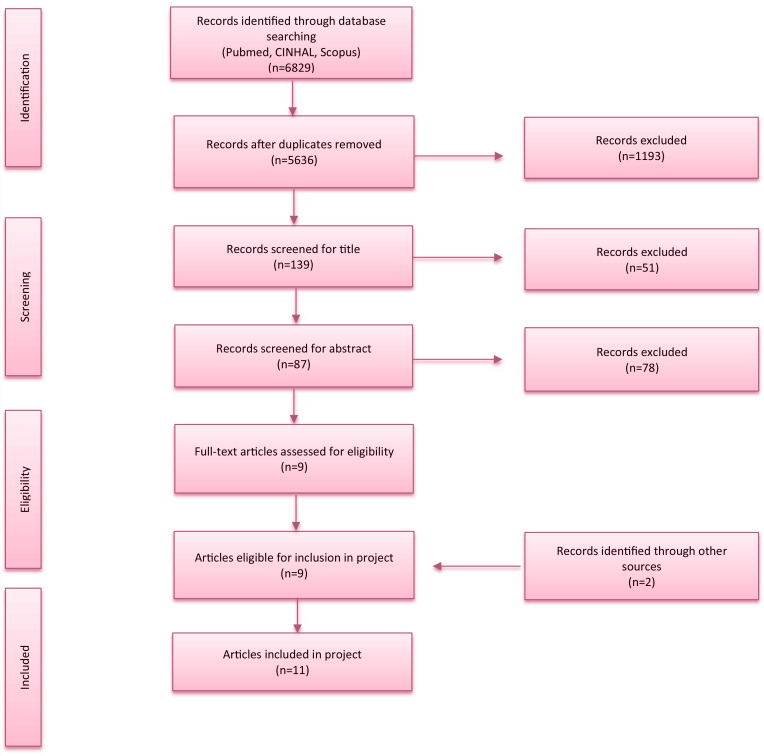
PRISMA flowchart for article selection

## RESULTS

Eleven publications were included in this scoping review, articles considered were published between 1992 and 2017. The majority of the studies were conducted in the United Kingdom (n=5), the NICE Guideline and the Guidance of the Glasgow City Council were included, both are English documents. One article was from the Netherlands. There are two United States based publications. In addition, there is an article from Canada and one from Australia. Type of articles and main findings are reported in Table 1. Evidence considered in this review is organised into themes and categories. Articles were evaluated through two different steps.

### First step

Relevant publications were appraised with the aim to identify a definition of vulnerability, and four themes emerged. Eight definitions were extracted from articles^9,18-21^. A deeper evaluation led to the identification of a key concept within each definition (Table 2). According to semantic and content affinities, checked and compared by three midwives, four themes emerged from these eight definitions and from key concepts associated with them:

**Table 2:** First step: definitions of vulnerability and key concepts

*N*	*Article and Year*	*Definition*	*Key concept*
I	Scupholme et al.^18^ 1992	Populations that are likely to experience poorer health outcomes due to their age, race/ethnicity, financial status, geographic location, and immigrant/migrant status.	Poorer outcomes than average
II	National Institute of Health and Clinical Excellence (NICE)^9^ 2010	Women considered to be vulnerable due to social and psychological difficulties that pose a potential risk to the foetus, infant and child.	Social and psychological difficulties
III	Menke et al.^19^ 2014	Women at risk of poor maternal and neonatal outcomes resulting from social disadvantages.	Poor maternal and neonatal outcome
IV	Glasgow Child Protection Committee^20^ 2008	Women with significant obstetric risks or women with complex social needs.	Obstetric risks; Complex social needs
V	De Groot et al.^21^ 2016	One or more complications (risk factors to foetal health: psychopathology, psychosocial problems and substance abuse) with lack of individual and/or social resources.	Risk factors for foetal health; Lack of individual and/or social resources
VI	Briscoe et al.^22^ 2016	Women who experience ‘threat’ from a physical, psychological or social perspective, where ‘barriers’ and ‘coping strategies’ conditions cause a status of vulnerability.	Threat
VII	MacMullen et al.^23^ 1992	Stress variables contributing to antenatal vulnerability. […] Hospitalisation is often a stressful experience. […] Women are vulnerable due to emotional and physical challenges that are often related to antenatal hospitalisation.	Emotional and physical challenges
VIII	MacMullen et al.^23^ 1992	The vulnerability emerges due to environmental and psychosocial factors.	Environmental and psychosocial factors

1. Poor health outcome or status, reported in seven definitions (I^18^, II^9^, III^19^, IV^20^, V^21^, VII^22^, VIII^23^);
2. Exposure to a risk reported in one definition (VI^22^);
3. Complex social needs reported in one definition (IV^20^);
4. Lack of resources reported in one definition (V^21^).

### Second step

Evidence was evaluated with the aim to identify which women are defined as vulnerable. Authors reported examples of social groups that were identified as vulnerable due to different reasons^9,18,19,24-26^. These social groups have been reported into six matrices, called matrices of vulnerable populations. Following a semantic and content analysis of the populations’ characteristics reported into the matrices, eleven themes emerged, with a consensus between three midwives. Themes were associated with features found in social groups identified as vulnerable (Table 3):

**Table 3:** Second step: matrices associated with social groups defined as vulnerable and themes

*N*	*Article and Year*	*Vulnerable social groups*	*Theme*
I	Tezcan et al.^24^ 2011	Ethnic minorities women; newly arrived migrants; refugee; asylum seekers; women with language difficulties; women from deprived communities.	Immigration AND language difficulties AND demographic characteristics AND lack of resources
II	Glasgow Child Protection Committee^20^ 2008	Asylum seekers; gender-based violence; would benefit from social work support; women who are resistant to professional intervention; learning difficulties that could impact on parenting; domestic violence with child protection issues; homeless; living in supported accommodation; young mothers; substance misuse; involvement in the criminal justice system; positive Human Immunodeficiency Virus (HIV+); child protection issues; mental health issues.	Immigration AND poor health status AND substance abuse AND involvement in criminal justice or being violence-prone AND care leaver AND family disruption AND homelessness AND demographic characteristics AND need of welfare/care service
III	Scupholme et al.^18^ 199	Poor women; adolescent; part of a minority ethnicity; immigrant status; living in medically unserved areas.	Demographic characteristics AND immigration AND lack of resources AND distance from care service
IV	National Institute of Health and Clinical Excellence (NICE)^9^ 2010	Substance misuse; domestic abuse; immigrants or asylum seekers or refugee or difficulties speaking or understanding English; aged <20 years; homelessness.	Substance abuse AND family disruption AND demographic characteristics AND immigration AND language difficulties AND homelessness
V	Menke et al.^19^ 2014	Young parents; women with substance misuse; past/current mental health issues; refugee and from ethnic minority.	Demographic characteristics AND immigration AND poor health status AND substance abuse
VI	Malebranche et al.^26^ 2017	Refugee women	Immigration

1. Immigration or language difficulties, reported in all six matrices^9,19,20,24,26;^
2. Demographic characteristics, reported in five matrices (I^24^, II^20^, III^18^, IV^9^, V^19^);
3. Substance abuse, reported in three matrices (II^20^, IV^9^, V^19^);
4. Poor health status or outcome reported in two matrices (II^20^, V^19^);
5. Lack of resources reported in two matrices (I^24^, III^18^);
6. Family disruption, reported in two matrices (II^20^, IV^9^);
7. Homelessness, reported in two matrices (II^20^, IV^9^);
8. Involvement in crimes or being violence-prone reported in one matrix (II^20^);
9. Care leaver reported in one matrix (II^20^);
10. The need for care or welfare care service reported in one matrix (II^20^);
11. Distance from care service reported in one matrix (III^18^).

Some of the eleven themes also arose from the first step of the articles’ assessment. Therefore, an analysis to merge both steps was required. Themes emerged from definitions of vulnerability, and the key concepts related to them, and these were combined with the themes that arose from the analysis of the matrices, thus leading to the identification of thirteen final themes:

1. Poor health outcome or status;
2. Lack of resources;
3. Demographic characteristics;
4. Immigration or language barrier;
5. Homeless;
6. Family disruption;
7. Exposure to a risk;
8. Need of care or welfare service;
9. Involvement in crimes or being violence prone;
10. Substance abuse;
11. Distance from care service;
12. Care leaver;
13. Complex social needs.

### Underpinning determinants of vulnerability concept

The final themes are characteristics related to women defined as vulnerable; therefore, they are referred to as indices of vulnerability. All the indices of vulnerability are shown in Table 4, and they are associated with the definition and matrix to which they refer. Semantic and content analysis, followed by a discussion between three midwives, allowed addressing the vulnerability indices into four main categories: Deficiency, Need, Risk Exposure, and Barriers. These categories are identified as four underpinning determinants of the vulnerability concept.

**Table 4:** Identification of indices of vulnerability emerged from First and Second step analyses

*Indices of vulnerability*	*Definition*	*Matrix*
Poor health outcome or status	I, II, III, IV, V, VII, VIII	
Lack of resources	V	I, III
Demographic characteristics		I, II, III, IV, V
Immigration or language barrier		I, II, III, IV, V, VI
Homeless		II, IV
Family disruption		II, IV
Exposure to a risk	VI	
Need for care or welfare service		II
Involvement in criminal justice or being violence prone		II
Substance abuse		II, IV, V
Distance from care service		III
Care leaver		II
Complex social needs	IV	

#### Deficiency

This determinant qualifies a vulnerable person who lacks material elements (indices: distance from care services, homeless, lack of resources) or alternatively who lacks nonmaterial elements (indices: poor health outcome or status, family disruption, care leaver); in both cases, these women are potentially at risk of worse health outcomes.

#### Need

This determinant qualifies a vulnerable person who may need additional care or financial support (indices: need of care or welfare service) or who needs additional social care (index: complex social needs).

#### Risk exposure

This determinant qualifies a vulnerable person who may have a higher exposure to threats or risk factors (index: exposure to risk).

#### Barriers

This determinant qualifies a person who has issues that interfere with normal interactions within the community, thus precluding benefits from social and community support. Women are often teenagers, immigrants or refugees, asylum-seekers, from a minority ethnic background or with a language barrier (indices: demographic characteristics, immigration or language difficulties). In some cases, barriers may be represented by a broad range of factors: women who misuse substances (index: substance abuse), women involved in crimes, or who are violence-prone (indices: involvement in crimes or being violence-prone).

The literature showed that some indices are more frequent than others. In addition, determinants originated from indices, and this could suggest that each determinant may contribute differently to the concept of vulnerability; the frequency of indices should be observed in order to recognise how determinants contribute to the concept of vulnerability. Within the articles included in this review, the most frequent indices of vulnerability were in order: poor health outcome or status, immigration or language difficulties, and demographic characteristics. These indices are associated with determinants of the vulnerability concept; among them, the most relevant are deficiency and barriers. Therefore, the vulnerability concept associated with pregnant women is based on four determinants, and it is mostly affected by Deficiency and Barriers.

## DISCUSSION

This scoping review identified 9 relevant publications, 1 Guideline and 1 Procedural guidance, spanning 25 years, involving works on the topic among different countries. This scoping review was conducted to explore the vulnerability concept and to establish a clear definition of this term in pregnancy. The analysis of the evidence allowed identifying the determinants underpinning the concept of vulnerability and drawing a definition of it. The vulnerability could be defined as a lack of health, related to the presence of at least one of the four determinants identified. Being vulnerable during the childbearing continuum is a concept that should also be associated with the complex system in which women live. As has been demonstrated^22^, the social context could play an important role in contributing to the vulnerability status of the women. This consideration gives the opportunity to interpret the vulnerability concept through the International Classification of Functioning, Disability and Health (ICF), developed by the World Health Organization^27^. The ICF is a classification of health and health-related domains. As the functioning and disability of an individual occur in a context, the ICF also includes a list of environmental factors. This classification system embraces the Activities and Participation component, which describes the person’s functional status, including communication, mobility, interpersonal interactions, selfcare, learning, applying knowledge etc. Each component of this Classification System is defined by qualifiers. The two qualifiers for the Activities and Participation component are the performance qualifier and the capacity qualifier. The performance qualifier describes what an individual does in his or her current context, including the environmental factors and all aspects of the physical, social and attitudinal spheres. The capacity qualifier, instead, describes an individual’s ability to execute a task or an action. This qualifier identifies the highest probable level of functioning that a person may reach; it reflects the environmentally adjusted ability of the individual. The vulnerability concept could be read throughout the Activities and Participation component of the ICF and should be regarded as the difference between the capacity and the performance qualifiers. The ICF helps to highlight once again the importance of the context. Social factors are as important as the biological ones for women’s health^1^. Given that vulnerability is highly context-specific, all healthcare professionals need to be able to determine the risks within the woman’s context. A woman who lacks a health determinant could be well supported in her own environment as, vice versa, apparently healthy women may have environmental risk exposure. Healthcare Systems should draw attention to the midwifery interventions that could play a role in improving health outcomes by limiting vulnerability^28^. Midwives have a key role in identifying vulnerable women as they work in primary healthcare and often approach marginalised mothers, so are well-positioned to make a difference in their outcomes^13^, achieving an appropriate plan of care that considers multidisciplinary interventions^9^.

### Strengths and limitations

This scoping review allowed us to explore what is currently known about the concept of vulnerability, which could be used to create a suitable tool to identify pregnant and postnatal vulnerable women. However, the volume of evidence was limited to nine articles, because the other documents were a Clinical Guideline and Procedural Guidance. Only articles in English were included, predominantly from the UK and the US. Although for this reason, results may not be generalizable to other countries, the rationale stated for the scoping review was that the vulnerability affecting pregnant women is an issue faced by women worldwide.

## CONCLUSIONS

The vulnerability status could be an important risk factor leading to poor pregnancy and infant outcomes. The vulnerability concept is dynamic and complex; girls and women face differential exposures and issues that are often poorly recognised. It appears crucial to offer these women appropriate midwifery care that involves a named midwife, as recommended by the NICE guideline. Midwives are the key to identify women who may be vulnerable, in order to ensure that they receive appropriate care. Mapping the existing services that provide additional care for these women should be the next step in order to improve the organisation of maternity services and perinatal health outcomes.

## FUNDING

There was no source of funding for this research.

## PROVENANCE AND PEER REVIEW

Not commissioned; externally peer reviewed.

## CONFLICTS OF INTEREST

The authors have completed and submitted the ICMJE Form for Disclosure of Potential Conflicts of Interest and none was reported.
